# Characterization of the complete mitochondrial genome of *Cryptotermes domesticus* (Blattodea: Kalotermitidae): Genome description and phylogenetic implications

**DOI:** 10.1002/arch.21974

**Published:** 2022-10-07

**Authors:** Guangyu Yu, Yufeng Cao, Peishan He, Weijun Li, Jianguo Wang

**Affiliations:** ^1^ Department of Plant Protection, Laboratory of Invasion Biology Jiangxi Agricultural University Nanchang Jiangxi China; ^2^ Department of Plant Protection, College of Biological Engineering Jiangxi Agricultural Engineering College Zhangshu Jiangxi China

**Keywords:** Kalotermitidae, *Cryptotermes*, mitochondrial genome, phylogenetic analysis

## Abstract

The complete mitochondrial genome of *Cryptotermes domesticus* (Haviland) was sequenced and annotated to study its characteristics and the phylogenetic relationship of *C. domesticus* to other termite species. The mitogenome of *C. domesticus* is a circular, close, and double‐stranded molecule with a length of 15,655 bp. The sequenced mitogenome contains 37 typical genes, which are highly conserved in gene size, organization, and codon usage. Transfer RNA genes (tRNAs) also have typical secondary structures. All of the 13 protein‐coding genes (PCGs) start with an ATN codon, except for *nad4*, which starts with GTG and terminates with the terminal codon TAA and TAG or the incomplete form T‐‐ (*cox2* and *nad5*). Most tRNAs have a typical cloverleaf structure, except for *trnS1*, in which this form is replaced by a simple loop and lacks the dihydrouridine (DHU) arm. The nucleotide diversity (Pi) and nonsynonymous (Ka)/synonymous (Ks) mutation rate ratios indicate that *nad1, cox1*, and *cox3* are the most conserved genes, and that *cox1* has the lowest rate of evolution. In addition, an 89 bp repeated sequence was found in the A + T‐rich region. Phylogenetic analysis was performed using Bayesian inference (BI) and maximum likelihood (ML) methods based on 13 PCGs, and the monophyly of Kalotermitidae was supported.

## Research highlights

- We sequence and analyze the complete mitochondrial genome of *Cryptotermes domesticus*.
- We analyze the genome size and nucleotide composition; codon usage, gene overlaps, and intergenic spacers; tRNA secondary structure, and the A + T‐rich region.
- The phylogenetic relationship of *Cryptotermes domesticus* among Kalotermitidae is clarified.

## Graphical abstract

This study sequenced and analyzed the complete mitochondrial genome of *Cryptotermes domesticus* and reveal its mitogenomic characteristics. Phylogenetic analysis was performed using Bayesian inference and maximum likelihood methods based on 13 protein‐coding genes, and the monophyly of Kalotermitidae was supported.

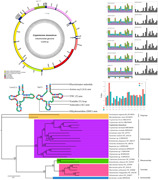

## 1 INTRODUCTION

The mitochondrial genome (mitogenome) is the most extensively studied genomic system in insects (Cameron, 2014). The mitogenome of insects may provide molecular evidence, thus allowing for phylogeny reconstruction and for inferences to be made regarding interspecific relationships (Xu et al., 2020). The insect mitogenome is typically a circular, closed, and double‐stranded DNA molecule that generally contains 37 genes. These genes include 2 ribosomal RNA genes (rRNAs), 13 protein‐coding genes (PCGs), 22 transfer RNA genes (tRNAs), and an A + T‐rich region (Boore, 1999; Cameron, 2014; Zhang & Hewitt, 1997).

Termite evolution is interesting due to termite diet diversity, social structures, and phenotypes (Bourguignon et al., 2014). Termites evolved from wood‐feeding cockroaches, and they form a sister group with the cockroach genus *Cryptocercus* (Li et al., 2017; Lo et al., 2000). The Kalotermitidae is the second largest termite family. The Kalotermitidae is monophyletic and the sister group of Neoisoptera, which comprises the Serritermitidae, Rhinotermitidae, and Termitidae (Bourguignon et al., 2014). *Cryptotermes* Banks (Blattodea: Kalotermitidae) include 69 living species and three fossil species (Casalla et al., 2016). *Cryptotermes* is a well‐known genus in the Kalotermitidae (Gay & Watson, 1982). *Cryptotermes* occur worldwide, mainly in tropical and subtropical zones, and include several species that are invasive and economically important pests (Gay & Watson, 1982). *Cryptotermes* nest and feed in dry wood. They can absorb a small amount of water from wood, absorb water from the atmosphere, and reabsorb water from feces (Gay & Watson, 1982). *Cryptotermes* can damage wooden structures, furniture, instruments, and even healthy living forest trees (Miller & Paton, 1983). Thompson et al. (2000) reconstructed the phylogenetic relationships of Australian lineages of Kalotermitidae based on cytochrome b (*Cytb*) and cytochrome oxidase II (*COII*) gene sequences. *Cryptotermes* represents the most apical lineage (Thompson et al., 2000). *C. domesticus* is an invasive termite that may have originated in Southeast Asia (Himmi et al., 2021). Because *C. domesticus* is cryptic, widely distributed, and destructive, previous studies have mostly focused on its biological characteristics. However, there is little information on the phylogeny and evolutionary biology of *C. domesticus*. Previous phylogenetic analyses mainly used morphological characters or were restricted to gene fragments. These studies suffer from relatively poor taxon sampling and an insufficient number of gene loci (Inward et al., 2007). Since these studies used different phylogenetic tree reconstruction methods, most trees have low node support values, or even different topologies, that are difficult to compare directly (Cameron et al., 2012). The taxonomic status and phylogenetic relationships of *C. domesticus* require further study based on additional molecular data. Therefore, analysis based on the mitogenome is a practical approach to study the phylogenetic relationships of these termites.

In this study, the complete mitogenome of *C. domesticus* was sequenced and analyzed. We analyzed the genome size and nucleotide composition; codon usage, gene overlaps, and intergenic spacers; tRNA secondary structure and the A + T‐rich region. The phylogenetic position of *C. domesticus* was reanalyzed based on Bayesian Inference (BI) and Maximum Likelihood (ML) methods in the Kalotermitidae. This study will help us understand the mitogenome evolution and phylogeny of the genus within Kalotermitidae.

## 2 MATERIALS AND METHODS

### 2.1 Sample collection

The specimens of *C. domesticus* were collected from Haikou, Hainan Province, China, on May 28, 2019. Some of the fresh specimens were preserved in 100% ethanol used for DNA extraction, the others were stored in 75% ethanol for morphology examination. All of the specimens were deposited in the Laboratory of Invasion Biology at Jiangxi Agricultural University, Nanchang, Jiangxi 340045, China.

### 2.2 DNA extraction, mitogenome sequencing, and assembly

Approximately 5 g of specimens were used for mtDNA extraction using an improved extraction method (Chen et al., 2011). After DNA extraction, 1 μg of purified DNA was fragmented and used to construct short‐insert libraries (insert size 430 bp) according to the manufacturer instructions (Illumina, San Diego, CA, USA), then the DNA was sequenced on an Illumina Hiseq. 4000 instrument (Borgström et al., 2011) (Shanghai BIOZERON Co., Ltd.). Before assembly, raw reads were first filtered. This filtering step was performed to remove the reads with adaptors, reads showing a quality score below 20 (Q < 20), reads containing a percentage of uncalled bases (“N” characters) equal to or greater than 10%, and duplicated sequences. The mitogenome was reconstructed using a combination of de novo and reference‐guided assemblies, and the following three steps were used to assemble mitogenomes. First, the filtered reads were assembled into contigs using SOAPdenovo2.04 (Luo et al., 2012). Second, contigs were aligned to the reference genome of *Cryptotermes* using BLAST, and aligned contigs (≥ 80% similarity and query coverage) were ordered according to the reference genome. Third, clean reads were mapped to the assembled draft mitochondrial genome to correct the wrong bases, and the majority of gaps were filled through local assembly.

### 2.3 Sequence annotation and analyses

The *C. domesticus* mitogenome was annotated using a pipeline of mitochondrial genome method. We used the tRNAscan‐SE search server and MITOS web server with default parameters to determine all tRNA genes and corresponding secondary structures (Bernt et al., 2013; Lowe & Eddy, 1997). The mitogenome of *C. havilandi* (GenBank: MW208858) was selected and used as a reference to annotate PCGs, rRNA genes, and the A + T‐rich region of *C. domesticus* in Geneious 8.1.3 (Kearse et al., 2012). The circular *C. domesticus* mitogenome map was drawn using OrganellarGenomeDRAW v1.2 (Lohse et al., 2007). The nucleotide composition and relative synonymous codon usage (RSCU) were analyzed using MEGA X software (Kumar et al., 2018). Strand asymmetry was calculated by the formulas: GC‐skew = [G − C]/[G + C] and AT‐skew = [A − T]/[A + T] (Perna & Kocher, 1995). The nucleotide diversity (Pi) and nonsynonymous (Ka)/synonymous (Ks) mutation rate ratios of 13 PCGs were analyzed using DnaSP v5.10.01 (Librado & Rozas, 2009).

### 2.4 Phylogenetic analysis

A total of 23 mitogenomes were used to construct the phylogenetic tree. Two species from Archotermopsidae and Hodotermitidae were selected as outgroups (Table 1). All mitogenome data were downloaded from GenBank (except *C. domesticus*). These mitogenome data were standardized and extracted for 13 protein‐coding genes (PCGs) using PhyloSuite v 1.2.2 (Zhang et al., 2020). All PCGs (excluding the stop codons) of the 25 termite species were aligned individually using codon‐based multiple alignments with MAFFT v7.313 software with default settings (Katoh & Standley, 2013). We removed the intergenic gaps and ambiguous sites using Gblocks v 0.91b software (Talavera & Castresana, 2007), and all PCGs genes were concatenated in PhyloSuite v 1.2.2 (Zhang et al., 2020). PartitionFinder2 software (Lanfear et al., 2016) was used to screen the optimal partition schemes and the best‐fit replacement models for constructing Bayesian inference (BI) and maximum likelihood (ML) trees using the branch lengths linked corrected AIC (AICc) model, the greedy search algorithm, and the gene and codon model. The results are presented in Table S1.

**Table 1:** Mitochondrial genome information used in this study

Family	Species	Length (bp)	GenBank accession No.	References
Kalotermitidae	*Cryptotermes brevis*	15674	MK618724	P. He et al. (2019a)
	*Cryptotermes declivis*	15678	MK599465	He et al. (2019b)
	*Cryptotermes domesticus*	15655	MT010558	This study
	*Cryptotermes havilandi*	15559	MW208858	Stiblik et al. (2021)
	*Cryptotermes secundus*	15695	KP026283	Bourguignon et al. (2014)
	*Glyptotermes satsumensis*	15611	KP026257	Bourguignon et al. (2014)
	*Glyptotermes* sp. A	15728	KP026263	Bourguignon et al. (2014)
	*Glyptotermes* sp. B	15101	KP026301	Bourguignon et al. (2014)
	*Glyptotermes* sp. C	15043	KP026300	Bourguignon et al., 2014
	*Rugitermes* sp.	15699	KP026284	Bourguignon et al. (2014)
	*Incisitermes minor*	15970	NC_037511	Liao et al. (2018)
	*Neotermes insularis*	15799	NC_018124	Cameron et al. (2012)
	*Neotermes* sp.	15401	KP026299	Bourguignon et al. (2014)
	*Neotermes koshunensis*	15589	NC_046741	Wang et al. (2019)
	*Roisinitermes ebogoensis*	15933	NC_040119	Scheffrahn et al. (2018)
Rhinotermitidae	*Coptotermes formosanus*	16326	NC_015800	Tokuda et al. (2012)
	*Reticulitermes chinensis*	15925	NC_025567	Chen et al. (2016)
Termitidae	*Odontotermes longignathus*	14950	NC_034130	Bourguignon et al. (2016)
	*Odontotermes obesus*	14950	NC_034027	Bourguignon et al. (2016)
	*Macrotermes barneyi*	15940	JX050221	Wei et al. (2012)
	*Macrotermes carbonarius*	14853	NC_034046	Bourguignon et al. (2016)
	*Macrotermes gilvus*	14950	NC_034110	Bourguignon et al. (2016)
Serritermitidae	*Serritermes serrifer*	14783	KP026264	Bourguignon et al. (2016)
Archotermopsidae	*Zootermopsis nevadensis*	15444	KJ958410	Qian (2016)
Hodotermitidae	*Microhodotermes viator*	15704	JX144931	Cameron et al. (2012)

BI and ML were used to construct the phylogenetic tree. MrBayes v 3.2.6 (Ronquist et al., 2012) and IQ‐TREE v.1.6.8 software (Minh et al., 2013; Nguyen et al., 2014) in PhyloSuite v 1.2.2 (Zhang et al., 2020), *Zootermopsis nevadensis* and *Microhodotermes viator* were used as outgroups. The ML phylogenetic analyses of clade support were assessed by 10,000 bootstrap pseudoreplicates with the ultrafast bootstrap of 1,000 replicates of the combined data set. In BI phylogenetic analyses, 2 million generations were run, with 25% of the generations as burn‐in. A PSRF close to 1.0 and a standard deviation of split frequencies below 0.01 were accepted.

## 3 RESULTS AND DISCUSSION

### 3.1 Mitogenome organization and nucleotide composition

**Figure 1:**
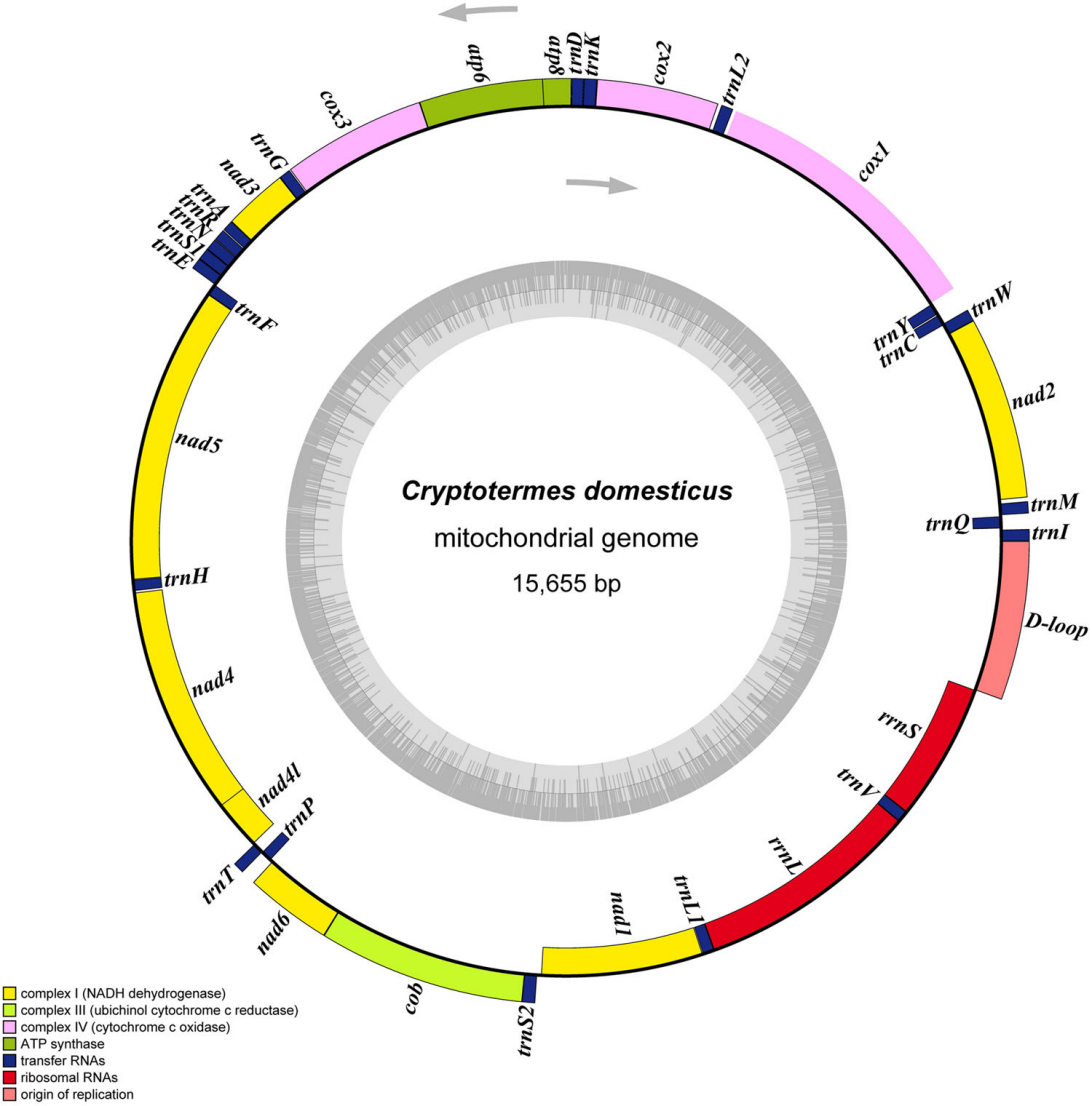
Circular map of the complete mitogenome of *Cryptotermes domesticus*. Different colors indicate different types of genes and regions. Genes in the outer circle are located on the J‐strand, and genes in the inner circle are located on the N‐strand.

The complete mitochondrial genome of *C. domesticus* is 15,655 bp long. It is a typical circular structure, close and double‐stranded, containing 13 PCGs, 22 tRNA genes, 2 rRNA genes and an A + T‐rich region (d‐loop) (Figure 1). The gene sequence is consistent with the original mitochondrial genome arrangement. The majority strand (J‐strand) encodes 23 genes including 9 PCGs and 14 tRNAs, and the minority strand (N‐strand) encodes the remaining 14 genes, including 4 PCGs, 8 tRNAs, and 2 rRNAs (Table 1). There are 21 intergenic spacers and 6 gene overlaps in the mitochondrial genome. The longest intergenic spacer is 23 bp between *trnS2*‐*nad1*, and the longest gene overlap is 7 bp between *nad4*‐*nad4l* (Table 2).

**Table 2:** Characteristics of the *Cryptotermes domesticus* mitogenome

Name	Strand	Start position	Stop position	Size (bp)	Start Codons	Stop Codons	Intergenetic nucleotides (bp)
*trnI*	J	1	65	65	‐	‐	8
*trnQ*	N	142	74	69	‐	‐	7
*trnM*	J	150	215	66	‐	‐	21
*nad2*	J	237	1238	1002	ATC	TAA	−1
*trnW*	J	1238	1304	67	‐	‐	8
*trnC*	N	1362	1297	66	‐	‐	5
*trnY*	N	1435	1368	68	‐	‐	1
*cox1*	J	1437	2981	1545	ATT	TAA	14
*trnL2*	J	2996	3062	67	‐	‐	18
*cox2*	J	3081	3747	667	ATA	T‐‐	0
*trnK*	J	3748	3818	71	‐	‐	0
*trnD*	J	3819	3885	67	‐	‐	0
*atp8*	J	3886	4044	159	ATA	TAA	7
*atp6*	J	4038	4718	681	ATG	TAA	−1
*cox3*	J	4718	5506	789	ATG	TAA	4
*trnG*	J	5511	5574	64	‐	‐	0
*nad3*	J	5575	5928	354	ATA	TAG	−1
*trnA*	J	5928	5989	62	‐	‐	3
*trnR*	J	5993	6057	65	‐	‐	1
*trnN*	J	6059	6125	67	‐	‐	0
*trnS1*	J	6126	6192	67	‐	‐	0
*trnE*	J	6193	6255	63	‐	‐	1
*trnF*	N	6322	6257	66	‐	‐	0
*nad5*	N	8048	6323	1726	ATG	T‐‐	1
*trnH*	N	8113	8050	64	‐	‐	10
*nad4*	N	9461	8124	1338	GTG	TAG	−7
*nad4l*	N	9742	9455	288	ATG	TAA	6
*trnT*	J	9749	9812	64	‐	‐	0
*trnP*	N	9877	9813	65	‐	‐	14
*nad6*	J	9892	10368	477	ATA	TAA	0
*cytb*	J	10369	11502	1134	ATG	TAG	2
*trnS2*	J	11501	11573	73	‐	‐	23
*nad1*	N	12535	11597	939	ATG	TAG	2
*trnL1*	N	12605	12538	68	‐	‐	0
*rrnL*	N	13929	12564	1366	‐	‐	0
*trnV*	N	13979	13912	68	‐	‐	0
*rrnS*	N	14784	13980	805	‐	‐	0
d‐loop	J	14785	15655	871	‐	‐	0

*Note*: N, N‐strand; J, J‐strand.

The nucleotide composition of *C. domesticus* is A = 41.8%, T = 24.9%, C = 21%, and G = 12.3%; and the A/T nucleotide composition is 66.7%. *C. domesticus* is similar to other insects in exhibiting a distinct A/T bias (Liu et al., 2018; Nie et al., 2020; Tang et al., 2020). In the whole mitogenome, the AT‐skew (0.25) is positive, and the GC‐skew (−0.26) is negative (Table S2). This situation has also been found in other Kalotermitidae mitogenomes.

### 3.2 Protein‐coding genes and codon usage

The total length of the 13 PCGs of *C. domesticus* includes 11,064 bp, accounting for 70.67% of the whole mitogenome. Among the PCGs, the smallest gene is *atp8* (159 bp), and the largest gene is *nad5* (1726 bp) (Tables 1 and S2). There are 9 PCGs on the J‐strand (*cox1, cox2, cox3, cytb, nad2, nad3, nad6, atp6, atp8*), and the other 4 PCG genes are on the N‐strand (*nad1, nad4, nad4l, nad5*). The whole 13 PCGs AT‐skew (−0.13) is negative, which reflects that the nucleotide composition of T is greater than A. GC‐skew (−0.01) means that the nucleotide composition of G and C were nearly equal. The A + T content of PCGs genes is lower in the Kalotermitidae (Table S3). Only *nad4* started with GTG while the other 12 PCGs started with ATN. Among the PCGs, only *cox2* and *nad5* terminated with T‐‐, while the others terminated with TAA (cox1, cox2, cox3, nad2, nad6, atp6, atp8, and *nad4l*) and TAG (*cytb, nad1, nad3*, and *nad4*). The incomplete termination codons are supposed to be completed through the rough polyadenylation processes and polycistronic transcription cleavage (Ojala et al., 1981).

**Figure 2:**
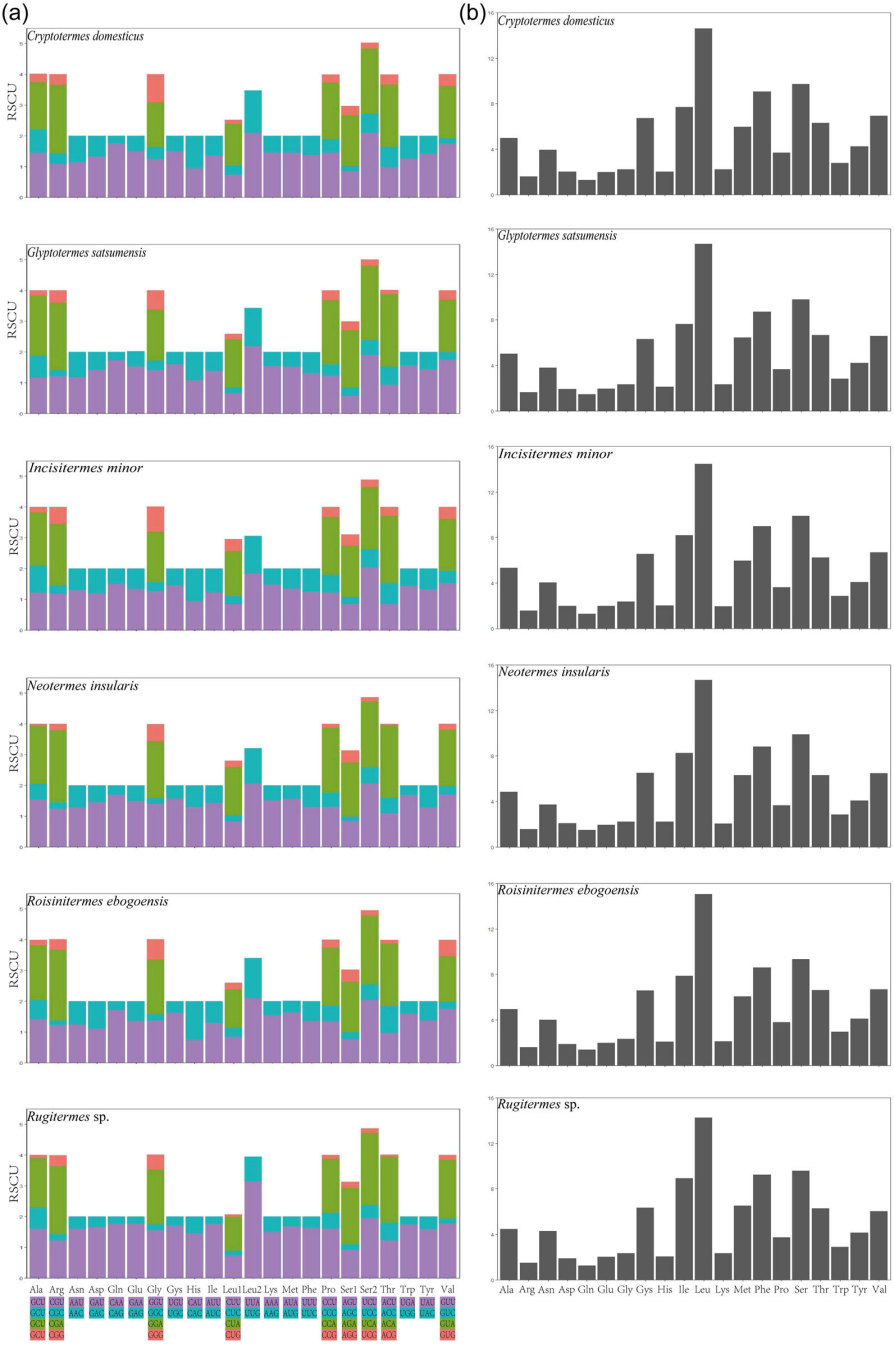
(a) Relative synonymous codon usage (RSCU) of six species of Kalotermitidae; (b) amino acid composition of six species of Kalotermitidae (the ordinate unit is percentage).

The six species of relative synonymous codon usage (RSCU) and amino acid composition were calculated (Figure 2). Leu, Ser, Phe, and Ile are the most frequently used amino acids. A total of 3,688 codons were found in 13 PCGs of *C. dometicus*. The most frequently used codons were UUA‐Leu (14.59%), UCA‐Ser (9.73%), UUU‐Phe (9.06%), and AUU‐Ile (7.7%) (Figure 2 and Table S4). These results suggest that UUA is the most preferred codon. Only the AT‐skew (0.02) was positive, indicating that the third position codon is rich in A and T.

### 3.3 Nucleotide diversity (Pi) and nonsynonymous (Ka)/synonymous (Ks) mutation rate ratios

**Figure 3:**
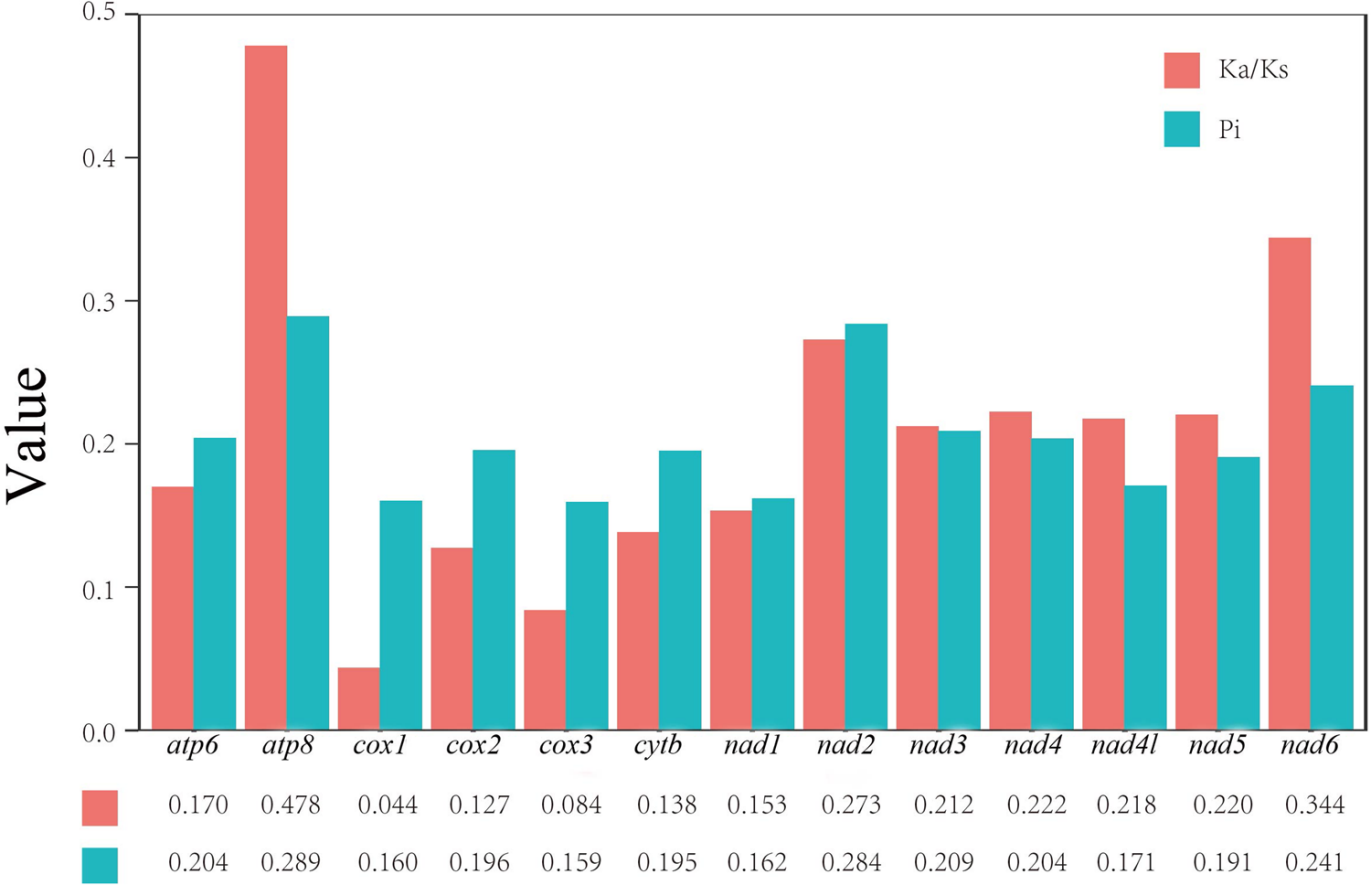
Nucleotide diversity (Pi) and nonsynonymous (Ka)/synonymous (Ks) mutation rate ratios of 13 protein‐coding genes (PCGs) of Kalotermitidae species (the Pi and Ka/Ks values of each PCG are shown under the gene name).

The nucleotide diversity (Pi) of six Kalotermitidae species were calculated (Figure 3). The values ranged from 0.159 (*cox3*) to 0.289 (*atp8*). Among the PCGs, *atp8* (0.289), *nad2* (0.284), and *nad6* (0.241) had the highest Pi values. The *nad1* (0.162), *cox1* (0.160), and *cox3* (0.159) had the lowest Pi values and were the most conserved genes.

The ratios of nonsynonymous (Ka)/synonymous (Ks) mutation rates were all below 1, suggesting that all PCGs are under purifying selection (Hurst, 2002). The *cox1* (0.044) gene has the lowest rate of evolution, *atp8* (0.478) and *nad6* (0.344) have relatively higher evolutionary rates. Species identification and delimitation in termites is extremely difficult. According to the analyses of Ka/Ks, the *cox1* is the best gene for use in DNA barcoding and preliminary phylogenetic relationships inference in Kalotermitidae. This finding will aid in the identification of termite species.

### 3.4 Transfer and ribosomal RNA genes

**Figure 4:**
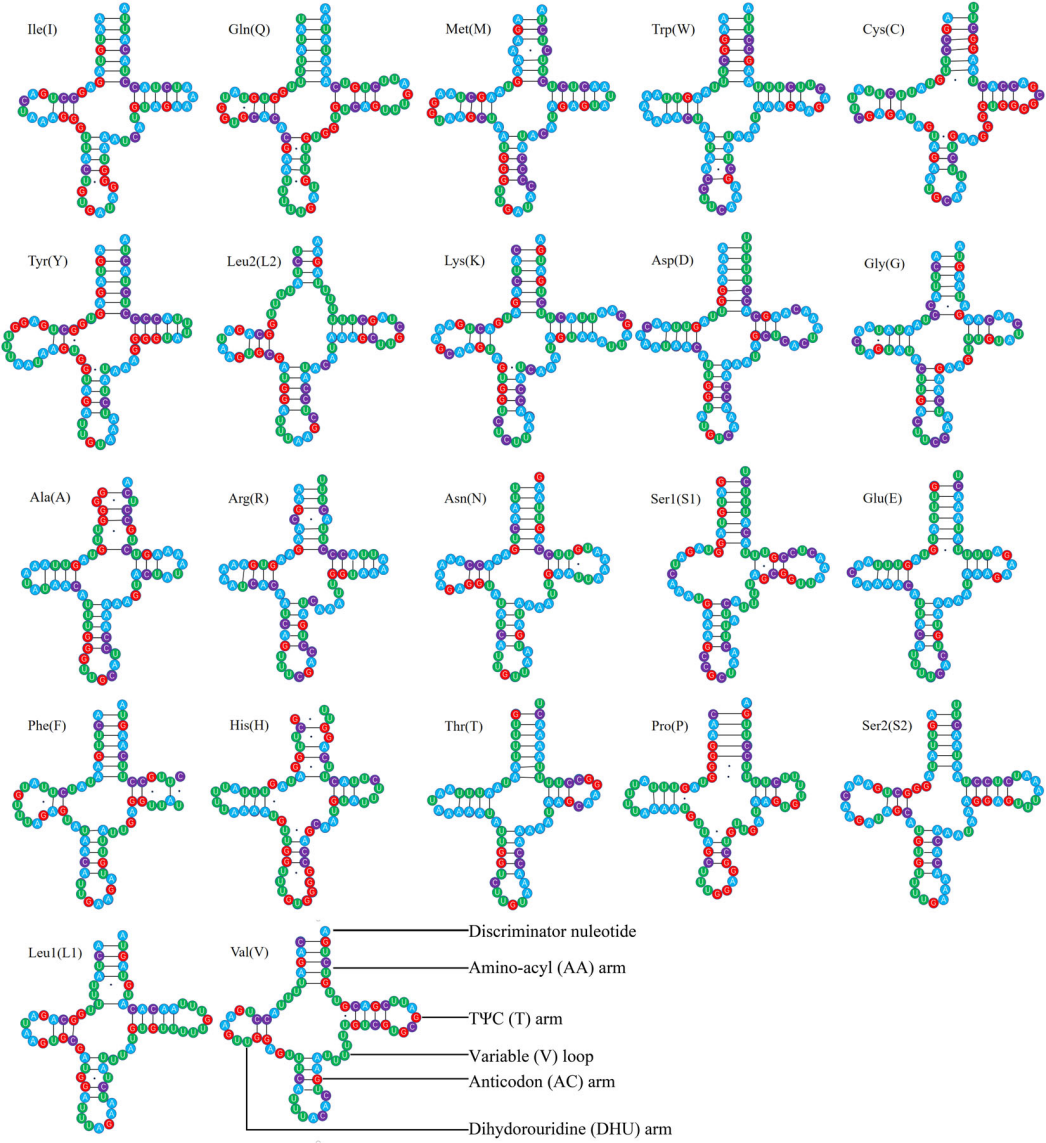
The secondary structures of the transfer RNA genes (tRNAs) in *Cryptotermes domesticus* mitogenome.

All 22 typical tRNAs were found in *C. domesticus* (Table 2). Their secondary structures are shown in Figure 4. The total length of tRNAs was 1459 bp (Table S2), ranging from 62 bp to 73 bp. The *trnS2* was the longest (73 bp), and the *trnA* was the shortest (62 bp) (Table 2). The AT‐Skew (0.03) and GC‐Skew (0.07) were all positive, indicating a slight bias toward A and G. There are 14 tRNA genes coded on the J‐strand (*trnI, trnM, trnW, trnL2, trnK, trnD, trnG, trnA, trnR, trnN, trnS1, trnE, trnT*, and *trnS2*), and the other 8 tRNA genes are coded on the N‐strand (*trnQ, trnC, trnY, trnF, trnH, trnP, trnL1*, and *trnV*) (Table 2). Most tRNAs have the typical cloverleaf structure (Figure 4), while *trnS1* lacks the dihydrouridine (DHU) arm and *trnF, trnR*, and *trnY* lack the TΨC arms. In *trnS1*, lack of the DHU arm is a common feature for most metazoan mitogenomes (Boore, 1999). Such abnormal tRNAs may sustain their function through a posttranscriptional RNA editing mechanism (Lavrov et al., 2000; Masta & Boore, 2004). Except for the classic base pairs (A‐U and C‐G), 18 noncanonical base pairs (A‐C and U‐G) were found in *trnI, trnQ, trnM, trnW, trnC, trnY, trnK, trnG trnA, trnR, trnN, trnS1, trnE, trnF, trnH, trnP, trnL1*, and *trnV*, and 4 other mismatched base pairs (U‐U and U‐C) were found in *trnQ, trnA, trnF*, and *trnL1*.

The *rrnL* (1,366 bp) and *rrnS* (805 bp) genes were encoded between *trnL1* and *trnV, trnV*, and D‐loop on the N‐strand (Figure 1). Because rRNA has no functional annotation features as PCGs, it is difficult to determine their boundaries (Ballard, 2000; Boore, 1999). The nucleotide composition of rRNA in *C. domesticus* is T (46.3%) > A (22.8%), and G (21.0%) > C (9.9%), with an AT‐Skew of −0.34 and GC‐Skew of 0.36 (Table S2).

### 3.5 A+T‐rich region

The A+T‐rich region acted in the initiation and regulation of replication and transcription in insects (Zhang & Hewitt, 1997; Zhang et al., 1995). The A + T‐rich region of *C. domesticus* is located between *rrnS* and *trnI* in the mitogenome. That total length of A + T‐rich region is 871 bp, A + T content is 68.7% with positive AT‐Skew (0.17) and negative GC‐Skew (−0.22) (Tables 2 and S2). In the A + T‐rich region, a sequence with 89 bp repeated twice from 8 bp to 267 bp, and a 9‐bp sequence was tandemly repeated twice.

### 3.6 Phylogenetic analysis

**Figure 5:**
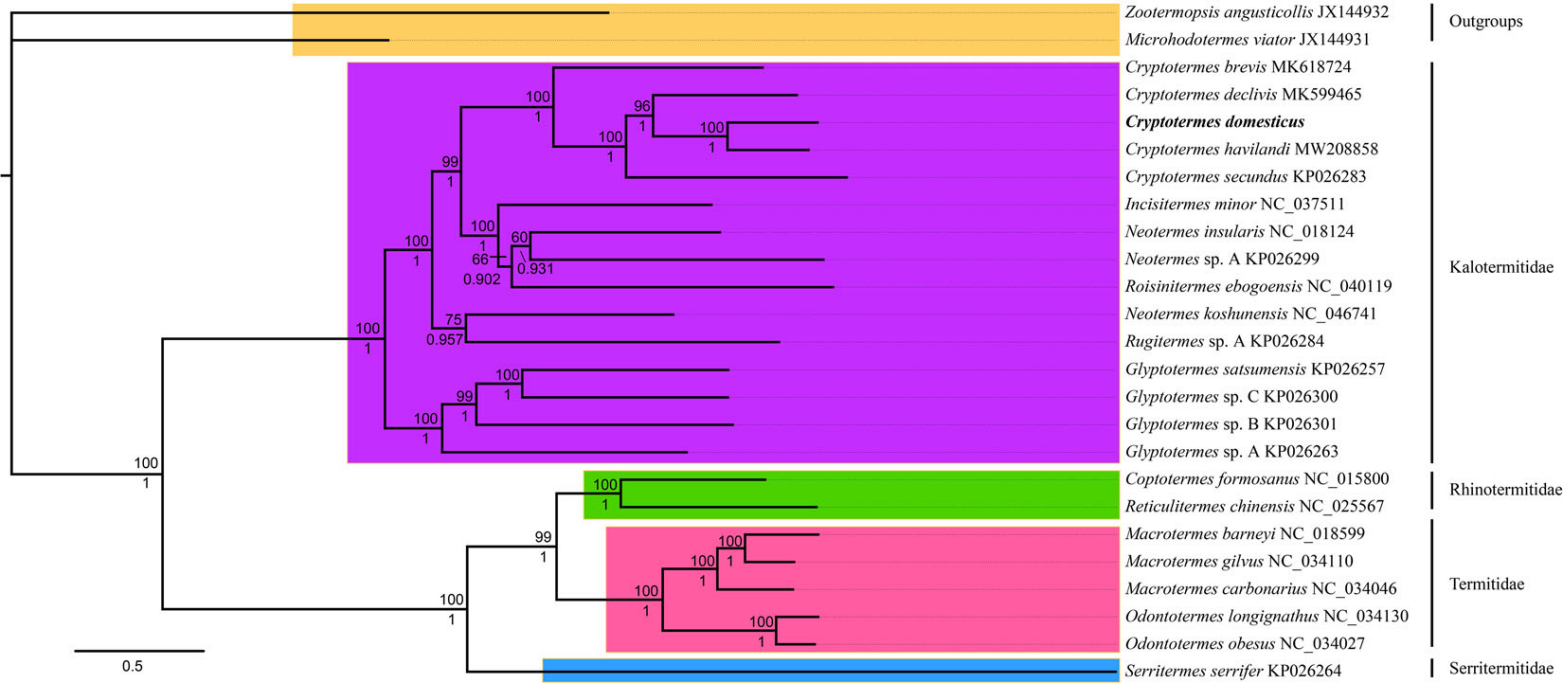
BI and ML analyses of the 13 protein‐coding genes (PCGs) produced similar tree topologies. The ML tree has the same topology as the BI tree, and their support values are reported above and below the nodes, respectively. The phylogram was constructed using the maximum likelihood method by 10,000 bootstrap pseudoreplicates with a standard bootstrap of 1000 replicates. BI, Bayesian inference; ML, maximum likelihood.

A phylogenetic tree of Kalotermitidae based on the 13 PCGs genes was constructed (Figure 5). The ML tree has the same topology as the BI tree, and their support values are reported above and below the nodes, respectively. The results indicate that Kalotermitidae is a monophyletic group and the sister group of Neoisoptera, which comprises Serritermitidae + Rhinotermitidae + Termitidae (Bourguignon et al., 2014). *Cryptotermes* represents the most apical lineage in Kaotermitidae and *C. brevis* is the most basal in *Cryptotermes* (Thompson et al., 2000). In this study, *Glyptotermes* was the most basal genus in the Kalotermitidae. This differs from the conclusions of Thompson et al. (2000), and suggests that more DNA data are needed to reveal the evolutionary position of *Glyptotermes. Neotermes koshunensis* is clustered with *Rugitermes* sp., but not with other *Neotermes*. Compared to the ML tree, the BI tree had higher support values about *Neotermes*. The genus *Neotermes* is not a monophyletic group and needs further study. The molecular data of other genera remain relatively poor. More molecular data are needed to understand the phylogenetic relationship of the genera within the Kalotermitidae.

## AUTHOR CONTRIBUTIONS

**Guangyu Yu**: Conceptualization (equal); data curation (equal); methodology (equal); software (equal); writing – original draft (lead). **Yufeng Cao**: Resources (equal); software (equal). **Peishan He**: Data curation (equal); resources (equal). **Weijun Li**: Data curation (equal); methodology (equal); software (equal); writing – review & editing (lead). **Jianguo Wang**: Conceptualization (equal); funding acquisition (lead); writing – review & editing (supporting).

## CONFLICTS OF INTEREST

The authors declare no conflicts of interest.

## Supporting information

Supplementary information.

Supplementary information.

Supplementary information.

## Data Availability

The data that support the findings of this study are openly available in [repository name e.g “figshare”] at [doi], reference number [reference number].
