# Supplementary material for: Characterization of the complete mitochondrial genome of Cryptotermes domesticus (Blattodea: Kalotermitidae): Genome description and phylogenetic implications
Source: Arch Insect Biochem Physiol. 2022 Oct 7;112(1):e21974. doi: 10.1002/arch.21974 (PMC10078508; doi:10.1002/arch.21974)
Supplement: Supplementary file 1 — Supplementary information. [file ARCH-112-0-s003.docx]

**Supplementary Data**

**Table S1.** The best-fit partitioning scheme and corresponding models used on phylogenetic tree.

| **Partition names** | **Sites** | **Best Model** |
| --- | --- | --- |
| charset Subset1 = 1-678\3 (*atp6* codon1) | 226 | GTR+G |
| charset Subset2 = 3047-3828\3, 2-678\3 (*cox3* codon2, *atp6* codon2) | 487 | TVM+I+G |
| charset Subset3 = 2367-3045\3, 3-678\3 (*cox2* codon3, *atp6* codon3) | 453 | K81UF+I+G |
| charset Subset4 = 10594-11076\3, 679-825\3 (*nad6* codon1*, atp8* codon1) | 210 | GTR+I+G |
| charset Subset5 = 680-825\3 (*atp8* codon2) | 49 | TVM+G |
| charset Subset6 = 681-825\3 (*atp8* codon3) | 49 | TRN+G |
| charset Subset7 = 826-2364\3 (*cox1* codon1) | 513 | SYM+I+G |
| charset Subset8 = 827-2364\3 (*cox1* codon2) | 513 | TVM+I+G |
| charset Subset9 = 828-2364\3 (*cox1* codon3) | 513 | TVM+I+G |
| charset Subset10 = 2365-3045\3, 3829-4959\3 (*cox2* codon1, *cytb* codon1) | 604 | GTR+I+G |
| charset Subset11 = 2366-3045\3, 3830-4959\3 (*cox2* codon2, *cytb* codon2) | 604 | GTR+I+G |
| charset Subset12 = 3046-3828\3 (*cox3* codon1) | 261 | SYM+I+G |
| charset Subset13 = 3048-3828\3, 3831-4959\3 (*cox3* codon3, *cytb* codon3) | 638 | K81UF+I+G |
| charset Subset14 = 7258-7542\3, 4960-5892\3 (*nad4l* codon1, *nad1* codon1) | 406 | GTR+I+G |
| charset Subset15 = 4961-5892\3 (*nad1* codon2) | 311 | TIM+I+G |
| charset Subset16 = 7260-7542\3, 4962-5892\3 (*nad4l* codon3, *nad1* codon3) | 406 | TVM+G |
| charset Subset17 = 6907-7257\3, 5893-6906\3 (*nad3* codon1, *nad2* codon1) | 455 | GTR+I+G |
| charset Subset18 = 5894-6903\3, 10595-11076\3 (*nad2* codon2, *nad6* codon2) | 499 | GTR+I+G |
| charset Subset19 = 5895-6906\3, (*nad2* codon3) | 338 | TVM+I+G |
| charset Subset20 = 6908-7257\3 (*nad3* codon2) | 117 | K81UF+I+G |
| charset Subset21 = 6909-7257\3, 10596-11076\3 (*nad3* codon3, *nad6* codon3) | 278 | HKY+G |
| charset Subset22 = 7259-7542\3 (*nad4l* codon2) | 95 | K81UF+G |
| charset Subset23 = 7543-8871\3, 8872-10593\3 (*nad4* codon1, *nad5* codon1) | 1017 | TVM+I+G |
| charset Subset24 = 7544-8871\3, 8873-10593\3 (*nad4* codon2, *nad5* codon2) | 1017 | GTR+I+G |
| charset Subset25 = 8874-10593\3, 75455-8871\3 (*nad5* codon3, *nad4* codon3) | 1017 | GTR+I+G |

**Table S2.** Nucleotide composition of *Cryptotermes domesticus* mitogenome.

| **Gene Region** | **Size (bp)** | **A(%)** | **T(%)** | **C(%)** | **G(%)** | **A+T(%)** | **G+C(%)** | **AT-Skew** | **GC-Skew** |
| --- | --- | --- | --- | --- | --- | --- | --- | --- | --- |
| Whole mitogenome | 15655 | 41.8 | 24.9 | 21.0 | 12.3 | 66.7 | 33.3 | 0.25 | -0.26 |
| 13 PCGs | 11064 | 28.5 | 37.2 | 17.4 | 16.9 | 65.7 | 34.3 | -0.13 | -0.01 |
| 1st coden | 3688 | 29.7 | 31.9 | 15.5 | 22.9 | 61.6 | 38.4 | -0.04 | 0.19 |
| 2st coden | 3688 | 18.7 | 44.2 | 21.1 | 16.0 | 62.9 | 37.1 | -0.41 | -0.14 |
| 3st coden | 3688 | 37.0 | 35.4 | 15.7 | 11.9 | 72.4 | 27.6 | 0.02 | -0.14 |
| tRNA genes | 1459 | 34.5 | 32.8 | 15.2 | 17.6 | 67.3 | 32.8 | 0.03 | 0.07 |
| rRNA genes | 2171 | 22.8 | 46.3 | 9.9 | 21.0 | 69.1 | 30.9 | -0.34 | 0.36 |
| D-loop | 871 | 40.1 | 28.6 | 19.2 | 12.2 | 68.7 | 31.4 | 0.17 | -0.22 |

Table S3. The A+T content of six species.

| Gene | A+T Content (%) | | | | | |
| --- | --- | --- | --- | --- | --- | --- |
|  | *Cryptotermes domesticus* | *Glyptotermes satsumensis* | *Rugitermes* sp*.* | *Incisitermes minor* | *Neotermes insularis* | *Roisinitermes ebogoensis* |
| Full genome | 67.76 | 67.49 | 70.44 | 65.44 | 67.83 | 66.64 |
| PCGs | 65.64 | 66.76 | 70.93 | 64.56 | 67.27 | 66.00 |
| tRNAs | 68.37 | 68.19 | 70.61 | 66.46 | 67.78 | 68.05 |
| 1^st^ condon position | 61.67 | 62.17 | 64.77 | 60.81 | 61.70 | 61.61 |
| 2^nd^ condon position | 62.86 | 62.63 | 62.88 | 62.68 | 62.86 | 62.80 |
| 3^rd^ condon position | 72.41 | 75.47 | 73.98 | 70.19 | 77.26 | 84.03 |
| *atp6* | 65.93 | 67.71 | 66.82 | 61.76 | 67.71 | 72.86 |
| *atp8* | 72.44 | 71.79 | 72.74 | 69.18 | 75.00 | 76.28 |
| *cox1* | 62.45 | 62.06 | 66.15 | 61.28 | 63.64 | 61.93 |
| *cox2* | 64.71 | 64.47 | 68.71 | 63.60 | 65.79 | 66.37 |
| *cox3* | 62.21 | 63.23 | 66.54 | 62.09 | 65.01 | 62.98 |
| *cytb* | 61.18 | 62.78 | 67.72 | 60.74 | 64.10 | 63.04 |
| *nad1* | 66.56 | 68.05 | 71.72 | 66.56 | 68.91 | 67.42 |
| *nad2* | 65.57 | 67.36 | 72.17 | 61.96 | 65.88 | 65.39 |
| *nad3* | 66.95 | 65.81 | 69.80 | 65.53 | 66.38 | 64.10 |
| *nad4* | 66.37 | 68.84 | 72.96 | 66.67 | 68.76 | 68.69 |
| *nad4L* | 71.58 | 74.04 | 76.14 | 71.99 | 71.93 | 68.78 |
| *nad5* | 69.57 | 69.97 | 73.74 | 67.88 | 70.26 | 69.04 |
| *nad6* | 68.78 | 72.36 | 76.63 | 70.58 | 71.17 | 69.94 |

**Table S4.** The RSCU status of *Cryptotermes domesticus,* *Glyptotermes satsumensis,* *Rugitermes* sp., *Incisitermes minor,* *Neotermes insularis,* and *Roisinitermes ebogoensis.*

| **Codon** | **Count** | **RSCU** | **Codon** | **Count** | **RSCU** | **Codon** | **Count** | **RSCU** | **Codon** | **Count** | **RSCU** |
| --- | --- | --- | --- | --- | --- | --- | --- | --- | --- | --- | --- |
| UUU (F) | 227/212/  277/206/  211/216 | 1.36/1.31/  1.62/1.24/  1.29/1.35 | UCU (S) | 94/86/  86/93/  94/88 | 2.09/1.90/  1.94/2.03/  2.05/2.03 | UAU (Y) | 111/111/  122/100/  96/105 | 1.41/1.42/  1.58/1.32/  1.27/1.38 | UGU (C) | 35/43/  39/34/  44/42 | 1.49/1.59/  1.70/1.45/  1.57/1.62 |
| UUC (F) | 107/111/  65/127/  116/103 | 0.64/0.69/  0.38/0.76/  0.71/0.65 | UCC (S) | 28/22  19/27/  25/22 | 0.62/0.48/  0.43/0.59/  0.54/0.51 | UAC (Y) | 46/45/  32/51/  55/47 | 0.59/0.58/  0.42/0.68/  0.73/0.62 | UGC (C) | 12/11/  7/13/  12/10 | 0.51/0.41/  0.30/0.55/  0.43/0.38 |
| UUA (L) | 187/197/  276/164/  187/195 | 2.09/2.18/  3.14/1.84/  2.06/2.10 | UCA (S) | 95/110/  104/93/  98/97 | 2.12/2.42/  2.34/2.03/  2.14/2.24 | UAA (*) | 0/0/  0/0/  0/0 | 0/0/  0/0/  0/0 | UGA (W) | 64/82/  93/75/  89/86 | 1.24/1.56/  1.74/1.42/  1.68/1.58 |
| UUG (L) | 124/112/  70/109/  104/121 | 1.38/1.24/  0.80/1.22/  1.15/1.30 | UCG (S) | 9/9/  7/11//  6/8 | 0.20/0.20/  0.16/0.24/  0.13/0.18 | UAG (*) | 0/0/  0/0/  0/0 | 0/0/  0/0/  0/0 | UGG (W) | 39/23/  14/31/  17/23 | 0.76/0.44/  0.26/0.58/  0.32/0.42 |
| CUU (L) | 65/59/  63/74/  74/77 | 0.72/0.65/  0.72/0.83/  0.82/0.83 | CCU (P) | 49/41/  55/41/  44/47 | 1.44/1.21/  1.59/1.22/  1.29/1.34 | CAU (H) | 35/42/  55/35/  53/28 | 0.93/1.08/  1.45/0.93/  1.29/0.73 | CGU (R) | 16/18/  17/17/  18/18 | 1.08/1.20/  1.21/1.17/  1.24/1.22 |
| CUC (L) | 28/17/  14/24/  18/29 | 0.31/0.19/  0.16/0.27/  0.20/0.31 | CCC (P) | 15/13/  18/19/  16/18 | 0.44/0.38/  0.52/0.57/  0.47/0.51 | CAC (H) | 40/36/  21/40/  29/49 | 1.07/0.92/  0.55/1.07/  0.71/1.27 | CGC (R) | 5/3/  3/4/  3/2 | 0.34/0.20/  0.21/0.28/  0.21/0.14 |
| CUA (L) | 121/141/  98/130/  143/115 | 1.35/1.56/  1.11/1.46/  1.58/1.24 | CCA (P) | 63/71/  61/63/  72/66 | 1.85/2.09/  1.77/1.88/  2.12/1.89 | CAA (Q) | 63/62/  66/55/  61/63 | 1.73/1.72/  1.76/1.49/  1.69/1.70 | CGA (R) | 33/33/  31/29/  34/34 | 2.24/2.20/  2.21/2.00/  2.34/2.31 |
| CUG (L) | 13/17/  7/35/  18/20 | 0.14/0.19/  0.08/0.39/  0.20/0.22 | CCG (P) | 9/11/  4//11/  4/9 | 0.26/0.32/  0.12/0.33/  0.12/0.26 | CAG (Q) | 10/10/  9/19/  11/11 | 0.27/0.28/  0.24/0.51/  0.31/0.30 | CGG (R) | 5/6/  5/8/  3/5 | 0.34/0.40/  0.36/0.55/  0.21/0.34 |
| AUU (I) | 191/194/  290/183/  217/189 | 1.35/1.38/  1.76/1.21/  1.42/1.30 | ACU (T) | 57/57/  70/49/  64/59 | 0.98/0.93/  1.21/0.85/  1.09/0.96 | AAU (N) | 83/82/  127/96/  88/92 | 1.14/1.17/  1.60/1.29/  1.28/1.24 | AGU (S) | 38/26/  40/39/  39/33 | 0.85/0.57/  0.90/0.85/  0.85/0.76 |
| AUC (I) | 93/88/  40/120/  89/102 | 0.65/0.62/  0.24/0.79/  0.58/0.70 | ACC (T) | 39/36/  34/39/  29/54 | 0.67/0.59/  0.59/0.68/  0.50/0.88 | AAC (N) | 62/58/  32/53/  50/56 | 0.86/0.83/  0.40/0.71/  0.72/0.76 | AGC (S) | 7/12/  9/10/  6/10 | 0.16/0.26/  0.20/0.22/  0.13/0.23 |
| AUA (M) | 160/181/  203/148/  182/182 | 1.45/1.51/  1.68/1.34/  1.56/1.63 | ACA (T) | 118/145/  124/126/  138/125 | 2.03/2.36/  2.14/2.18/  2.36/2.04 | AAA (K) | 59/67/  65/53/  57/61 | 1.44/1.54/  1.49/1.47/  1.50/1.54 | AGA (S) | 74/85/  81/76/  81/71 | 1.65/1.87/  1.83/1.66/  1.77/1.64 |
| AUG (M) | 60/58/  39/73/  52/42 | 0.55/0.49/  0.32/0.68/  0.44/0.38 | ACG (T) | 19/8/  4/17/  3/7 | 0.33/0.13/  0.07/0.29/  0.05/0.11 | AAG (K) | 23/20/  22/19/  19/18 | 0.56/0.46/  0.51/0.53/  0.50/0.46 | AGG (S) | 14/13/  9/17/  18/17 | 0.31/0.29/  0.20/0.37/  0.39/0.39 |
| GUU (V) | 110/106/  99/94/  102/108 | 1.73/1.74/  1.77/1.52/  1.70/1.74 | GCU (A) | 67/54/  66/59/  69/65 | 1.46/1.16/  1.60/1.20/  1.54/1.42 | GAU (D) | 49/50/  57/43/  56/38 | 1.32/1.41/  1.65/1.18/  1.45/1.10 | GGU (G) | 77/82/  91/76/  85/83 | 1.24/1.4/  1.55/1.26/  1.40/1.37 |
| GUC (V) | 12/15/  9/25/  16/15 | 0.19/0.25/  0.16/0.40/  0.27/0.24 | GCC (A) | 35/33/  29/44/  23/28 | 0.76/0.71/  0.70/0.89/  0.51/0.61 | GAC (D) | 25/21/  12/30/  21/31 | 0.68/0.59/  0.35/0.82/  0.55/0.90 | GGC (G) | 24/18/  11/18/  11/12 | 0.39/0.31/  0.19/0.30/  0.18/0.20 |
| GUA (V) | 108/104/  107/105/  110/92 | 1.69/1.7/  1.91/1.69/  1.83/1.48 | GCA (A) | 70/91/  66/85/  84/82 | 1.52/1.96/  1.60/1.73/  1.88/1.79 | GAA (E) | 61/66/  77/58/  61/58 | 1.49/1.53/  1.77/1.33/  1.49/1.35 | GGA (G) | 90/97/  105/99/  112/108 | 1.45/1.66/  1.79/1.64/  1.85/1.78 |
| GUG (V) | 25/19/  9/24/  12/33 | 0.39/0.31/  0.16/0.39/  0.20/0.53 | GCG (A) | 12/8/  4/9/  3/8 | 0.26/0.17/  0.10/0.18/  0.07/0.17 | GAG (E) | 21/20/  10/29/  21/28 | 0.51/0.47/  0.23/0.67/  0.51/0.65 | GGG (G) | 57/37/  28/49/  34/40 | 0.92/0.63/  0.48/0.81/  0.56/0.66 |
