# Supplementary material for: Characterization of the complete mitochondrial genome of Cryptotermes domesticus (Blattodea: Kalotermitidae): Genome description and phylogenetic implications
Source: Arch Insect Biochem Physiol. 2022 Oct 7;112(1):e21974. doi: 10.1002/arch.21974 (PMC10078508; doi:10.1002/arch.21974)
Supplement: Supplementary file 2 — Supplementary information. [file ARCH-112-0-s002.pdf]

Certificate of English Language Editing

Manuscript Title:

Characterization of the complete mitochondrial genome of Cryptotermes domesticus (Blattodea: Kalotermitidae): genome description and phylogenetic implications

Date of Revision:

September 8, 2022

Abstract:

The complete mitochondrial genome of Cryptotermes domesticus (Haviland) was sequenced and annotated to study its characteristics and the phylogenetic relationship of Cryptotermes domesticus to other termite species. The mitogenome of C. domesticus is a circular, close, and double-stranded molecule with a length of 15,655 bp. The sequenced mitogenome contains the 37 typical genes, which are highly conserved in gene size, organization, and codon usage. Transfer RNA genes (tRNAs) also have typical secondary structures. All of the 13 protein-coding genes (PCGs) start with an ATN codon, except for nad4, which starts with GTG and terminates with the terminal codon TAA and TAG or the incomplete form T-- (cox2 and nad5). Most tRNAs have a typical cloverleaf structure, except for trnS1, in which this form is replaced by a simple loop and lacks the dihydrouridine (DHU) arm. The nucleotide diversity (Pi) and nonsynonymous (Ka)/synonymous (Ks) mutation rate ratios indicate that nad1, cox1, and cox3 are the most conserved genes, and that cox1 has the lowest rate of evolution. In addition, a 89-bp...

This document certifies that the manuscript listed above was copy edited for English language by LetPub, with regard to grammar, punctuation, spelling, and clarity. All of our language editors are native English speakers with long-term experience in editing scientific and technical manuscripts. We are committed to leveling the playing field for researchers whose native language is not English.

- Documents receiving this certification should be regarded as having undergone professional editorial revision for English language before submission. However, the authors may accept or reject LetPub's suggestions and changes at their own discretion and LetPub does not have editorial control over the submitted documents.
- The language quality of the submitted document is the sole responsibility of the submitting authors subject to those authors' adherence to LetPub's revisions and instruction. LetPub's provision of service does not constitute a guarantee or endorsement of the authors' work herein.
- Neither the research content nor the authors' intended meaning were altered in any way during the editing process.
- If you have any questions or concerns about this edited document, please contact us at support@letpub.com

LetPub
Your Manuscript Editor

LetPub is an author service brand owned and operated by Accdon LLC. Headquartered in the Boston area, we are a full-spectrum author services company with a large team of US-based certified language and scientific editors, ISO 17001 accredited translators, and professional scientific illustrators and animators. We advocate ethical publication practices and are an official member of the Committee on Publication Ethics (COPE).

For more information about our company, services, and partnership programs, please visit www.letpub.com.

© 2022 Accdon, LLC. All Rights Reserved. Tel: 1-781-202-9968 Email: info@accdon.com Address: 400 Fifth Ave, Suite 530, Waltham, MA 02451, United States
