# Supplementary material for: Characterization of the complete mitochondrial genome of Cryptotermes domesticus (Blattodea: Kalotermitidae): Genome description and phylogenetic implications
Source: Arch Insect Biochem Physiol. 2022 Oct 7;112(1):e21974. doi: 10.1002/arch.21974 (PMC10078508; doi:10.1002/arch.21974)
Supplement: Supplementary file 3 — Supplementary information. [file ARCH-112-0-s001.docx]

**Sep 8, 2022**

**Dear editor,**

Thank you for giving us the opportunity to submit a revised manuscript, entitled “**Characterization of the complete mitochondrial genome of *Cryptotermes domesticus* (Blattodea: Kalotermitidae): genome description and phylogenetic implications**” for publication in the ***Archives of Insect Biochemistry & Physiology***. We appreciate the effort of the reviewers to provide feedback and the insightful comments on our manuscript. Herein, we have incorporated all of the suggestions made by the reviewers. Those changes are highlighted in red for a point-by-point response to the reviewers’ comments and concerns. All line numbers refer to the revised manuscript file with tracked changes. This revision has been double-checked by a native English expert, and we have also provided the certificate.

Sincerely,

Prof. Jianguo Wang

Laboratory of Invasion Biology, Jiangxi Agricultural University,

Nanchang, Jiangxi 340045, China

## Reviewing: 1

Comments to the Author

This is a revised manuscript that describes a newly sequenced mitochondrial genome. The current version is much improved. However, many statement issues, language errors, and incomplete descriptions remain. I suggest the authors find an english-speaking expert in mitochondrial genome to revise the manuscript. Below, I pointed out some of the issues.

Reply: Thank you for your kind comments on our study. This revision has been double-checked by a native English expert, and we have also provided the certificate. In the revised manuscript, we have addressed all your suggestions, please also see our point-by-point reply bellow.

1. mitogenome is the abbreviation of the mitochondrial genome; it should not be used as a keyword; define mitogenome before the first used.

Reply: Thank you for your suggestion. We have made adjustments in the abstract and foreword, line 23, 25.

2. Line 53-56, the introduction to the phylogenetic study is limited. Add more results and point out the current problem in the phylogenetic relationships of termites.

Reply: Thank you for your comment. We have added relevant contentin introduction, line 52-59.

3. Line 94, MW209958, is this a GenBank accession number?

Reply: Yes. We have revised it into (GenBank: MW208858), line 96.

4. Line 109, codon is not a model of MAFFT. This issue was not resolved in this version.

Reply: Thank you for your kind comment. We have revised them in the manuscript, line 111-113.

5. Lines 112-113, the authors did not state how the data were partitioned. By gene, by codon, or by gene and codon?

Reply: By gene and codon, we have revised them in the manuscript, line 118.

6. Line 116, selected? used?

Reply: We have revised it into used, line 386.

7. Line 117, implemented, or used?

Reply: We have revised it into used, line 120.

8. Line 119, Lines 120-121, corrected the grammar errors.

Reply: Thank you for your comment, we have revised it, line 120-123.

9. In the phylogenetic analysis method section, many parameters were missed. I suggest the authors add detailed method of phylogenetic inferences.

Reply: Thank you for suggestion. We have added it in line 123-124.

10. Line 139, were the AT skew and GC skew normal in this mitochondrial genome?

Reply: Yes.

11. Table 2, Size(bp), Size (bp); From, Start position; To, Stop position

Reply: We have revised it.

12. Figure 1, the gene names were too small in font size. Add annotation of figure 1.

Reply: Thank you for your kind comment. We have made relevant adjustments, line 394-396.

Line 222, 5., 5

Reply: Thank you for your kind comment. We have revised them in the manuscipt.

Figure 5, add figure legend. What are the values on the nodes represent?

Reply: Thank you for your comment. We have added notes, line 414-417.

Line 224, and the sister group... This is an incomplete sentence.

Reply: Thank you for your comment. We have revised this sentence, line 210-212.

## Reviewing: 2

Comments to the Author

Line 10- 13: “the mitogenome…structures too.” Need to be divided into 2 sentences.

Reply: Thank you for your comment. We have divided that, line 10-13.

Line20-21: “the results support Kalotermitidae is a monophyletic group” change to “and the monophyly of Kalotermitidae was supported.”

Reply: Thank you for your kind comment. We have revised them in the manuscript, line 21-22.

Line39: “are one of”?

Reply: Thank you for your kind comment. We have revised them in the manuscript, line 39-40.

Line 55: “DNA data” change to “molecular data”

Reply: Thank you for your kind comment. We have revised them in the manuscript, line 58.

Line 148 “PCGs” Provide full name when abbreviation first appears

Reply: Thank you for your kind comment. We have provided full name at line 30.

Line 186-187 : make sure the consistency of tense.

Reply: Thank you for your kind comment. We have revised them in the manuscipt.
